# Prevalence and Identification of Livestock Tick by Sex Ratio and Host in Tehran Province

**DOI:** 10.1002/vms3.70702

**Published:** 2025-11-18

**Authors:** Ebrahim Abbasi

**Affiliations:** ^1^ Research Center for Health Sciences Institute of Health Shiraz University of Medical Sciences Shiraz Iran; ^2^ Department of Medical Entomology and Vector Control School of Health Shiraz University of Medical Sciences Shiraz Iran

**Keywords:** ectoparasites, host association, *Rhipicephalus sanguineus*, sex ratio, Tehran Province

## Abstract

**Background:**

Ticks are major ectoparasites affecting livestock health and productivity. Their sex ratio and host specificity influence pathogen transmission.

**Objective:**

To identify tick species and determine sex ratios across different host animals in Tehran Province, Iran.

**Methods:**

Ticks are one of the main risk factors in transmitting pathogens to livestock and humans. From a total of 1623 animals, 806 ticks were collected, of which 685 were hard ticks and 121 were soft ticks. Ticks were sexed and identified using morphological keys.

**Results:**

Females comprised 57.99% and males 42.01% of ticks. Sheep had the highest infestation rate (60.04%), cattle the lowest (0.62%). *Rhipicephalus sanguineus* sensu lato was the most prevalent species (39.96%).

**Conclusion:**

High sheep infestation and dominance of *R. sanguineus* highlight the need for targeted tick control to reduce disease risks and economic loss in livestock.

## Graphical abstract

This study identified tick species infesting livestock and poultry in Tehran Province, revealing a female‐biased sex ratio and sheep as the most infested hosts. *Rhipicephalus sanguineus* sensu lato was the dominant species. These findings underscore the need for host‐targeted tick control to mitigate disease transmission and economic losses.

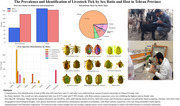

## 1 Introduction

Generally, ticks are divided into two large families, including Ixodidae (hard ticks) and Argasidae (soft ticks), with diverse species. Ticks are critical ectoparasites that play a significant role in transmitting pathogens. These blood‐sucking ectoparasites, which are also known as pathogenic vectors, transmit bacteria, viruses, and protists to their hosts, including animals and humans. These pathogens cause various types of diseases, such as bacterial diseases (Q fever, Lyme disease, relapsing fever, and borreliosis), fungal diseases (dermatophilosis), protozoal diseases (babesiosis and theileriosis), and rickettsial diseases (ehrlichiosis, Brazilian spotted fever, anaplasmosis, and Rocky Mountain spotted fever) (Abbasi 2024; Abbasi 2025a; Abbasi et al. 2025).

Since sex ratio is a critical parameter determining the status and dynamics of animal populations, studies on sex ratio are vital for understanding the biology of populations and the biology of pathogens. Accordingly, arthropod vectors (e.g., ticks) and sex ratio could play different roles in pathogen transmission. Although ticks have been known since time immemorial, their role in causing livestock troubles was initiated in the mid‐19th century. Due to the growth of the world's population and nutritional needs, the number of livestock has increased rapidly throughout the industry. At the same time, concerns and issues related to ticks have emerged (Abbasi et al. 2023; de la Fuente et al. 2008; Ghahvechi Khaligh et al. 2021; Randolph 2004).

In 1814, piroplasmosis was diagnosed in cattle in the United States. In 1821, it was discovered that the disease was transmitted to cattle by the ticks' bite called *Rhipicephalus (Boophilus) annulatus* (Rizk et al. 2022). In 1971, Mazloum conducted studies on the geographical distribution, seasonal activity, preferred tick hosts, and diseases transmitted to livestock and humans (Mazloum 1971).

Pourmand et al. have also conducted a study to determine the frequency and species diversity of hard ticks and their sex ratio in equids in Sardasht suburb, West Azerbaijan Province, Iran. The results show that 85.48% of the ticks were male and 14.51% were female, with the highest frequency of *Hyalomma anatolicum* (67.74%), *Rhipicephalus bursa* (21.94%), *H. marginatum* (8.01%), and *Dermacentor marginatus* (2.29%), respectively (Pourmand et al. 2021).

It is important to note that the tick species *Rhipicephalus sanguineus* has historically been referred to as a single species. However, recent molecular and taxonomic studies have demonstrated that this name encompasses a complex of genetically distinct but morphologically similar species, now collectively referred to as *R. sanguineus* sensu lato. In this study, ticks were identified and reported using this updated nomenclature to align with current scientific understanding and ensure taxonomic accuracy (Dantas‐Torres et al. 2013).

Since tick bites can transmit diseases to livestock and poultry, it is important to identify the most common ticks based on the host and the sex ratio of the ticks, which can be a practical way to combat ticks and prevent the spread of diseases, ultimately minimising economic losses due to livestock. Thus, this study aimed to determine the sex and identify ticks in different hosts, including camels, sheep, cattle, dogs, chickens, and pigeons in Tehran Province in 2019. Although several studies have addressed tick distribution in various provinces of Iran, limited data exist for Tehran Province, particularly concerning sex ratios and host‐specific infestations. This study aims to fill that gap by identifying tick species, comparing their sex distribution, and evaluating host preferences. Hypothesis: Tick species and sex ratios differ significantly across livestock hosts in Tehran Province, influenced by environmental and host‐related factors (Abbasi et al. 2022; Abbasi et al. 2019; Azari‐Hamidian and Harbach 2023).

## 2 Materials and Methods

### 2.1 Geographical Area

**FIGURE 1:**
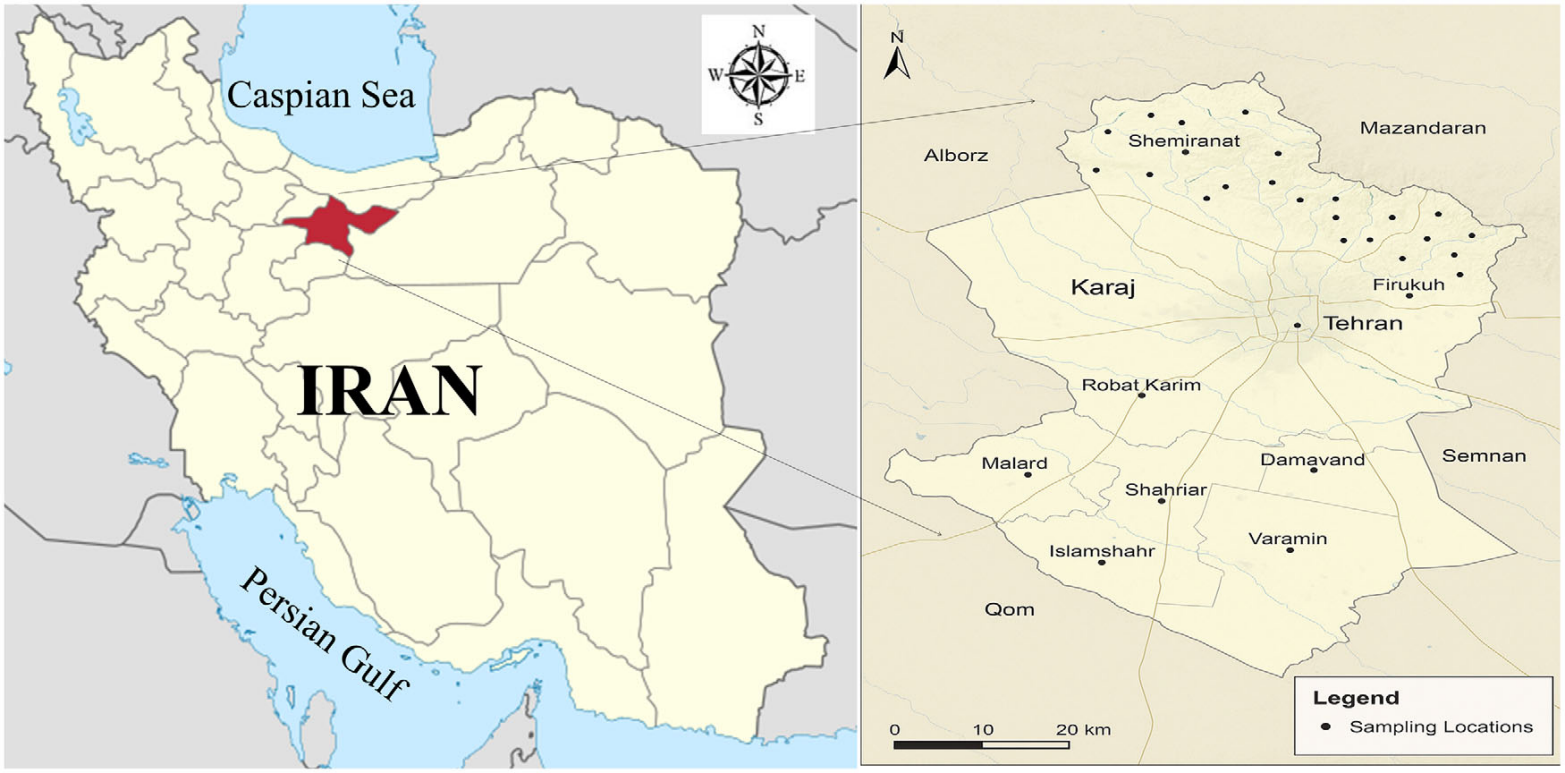
Selected areas of Tehran province for sampling.

This study was performed in 20 selected villages of Tehran Province with an area of approximately 185.956 km^2^, located between 34° and 5.36° north latitude and 50° and 53° east longitude (Figure 1).

### 2.2 Sampling Size and Method

The sample size was determined using the (Hosseini‐Vasoukolaei et al. 2014) procedure (*d* = 0.045, *p* = 0.3, (1 – *p*) = 0.7). Accordingly, 685 hard ticks and 121 soft ticks were collected from 1623 livestock and poultry. Data were analysed using SPSS version 25. Chi‐square tests were applied to assess significant differences in tick prevalence among host species and locations, and to evaluate the male‐to‐female sex ratio. A *p*‐value of less than 0.05 was considered statistically significant (Abbasi 2025b; Abbasi 2025; Abbasi 2025c; Abbasi 2025d; Abbasi 2025e).

$$n_{0}=\frac{Z\frac{2}{1-\frac{\alpha }{2}}p\left(1-p\right)}{d^{2}}.$$

### 2.3 Sample Collection and Identification

Ticks were manually collected from infested livestock between March and September 2019, covering both warm and moderate seasons to assess seasonal variation in tick infestation. Sampling was conducted across 20 villages in Tehran Province, selected to represent both mountainous and plain ecosystems. In each village, approximately 80 animals including cattle, sheep, goats, poultry, dogs, and camels were sampled based on owner consent and accessibility, yielding a total of 450 examined animals (150 per species). Tick removal was performed by trained personnel using blunt‐tipped forceps or fine‐tipped tweezers to ensure safe extraction without damaging the tick or causing injury to the host. Animals were gently restrained without sedation, and sampling procedures were conducted under the supervision of a veterinary technician in accordance with ethical standards for ectoparasite surveillance. Collection focused on common tick attachment sites, such as the ears, neck, underbelly, groin, tail base, and between the legs.

**FIGURE 2:**
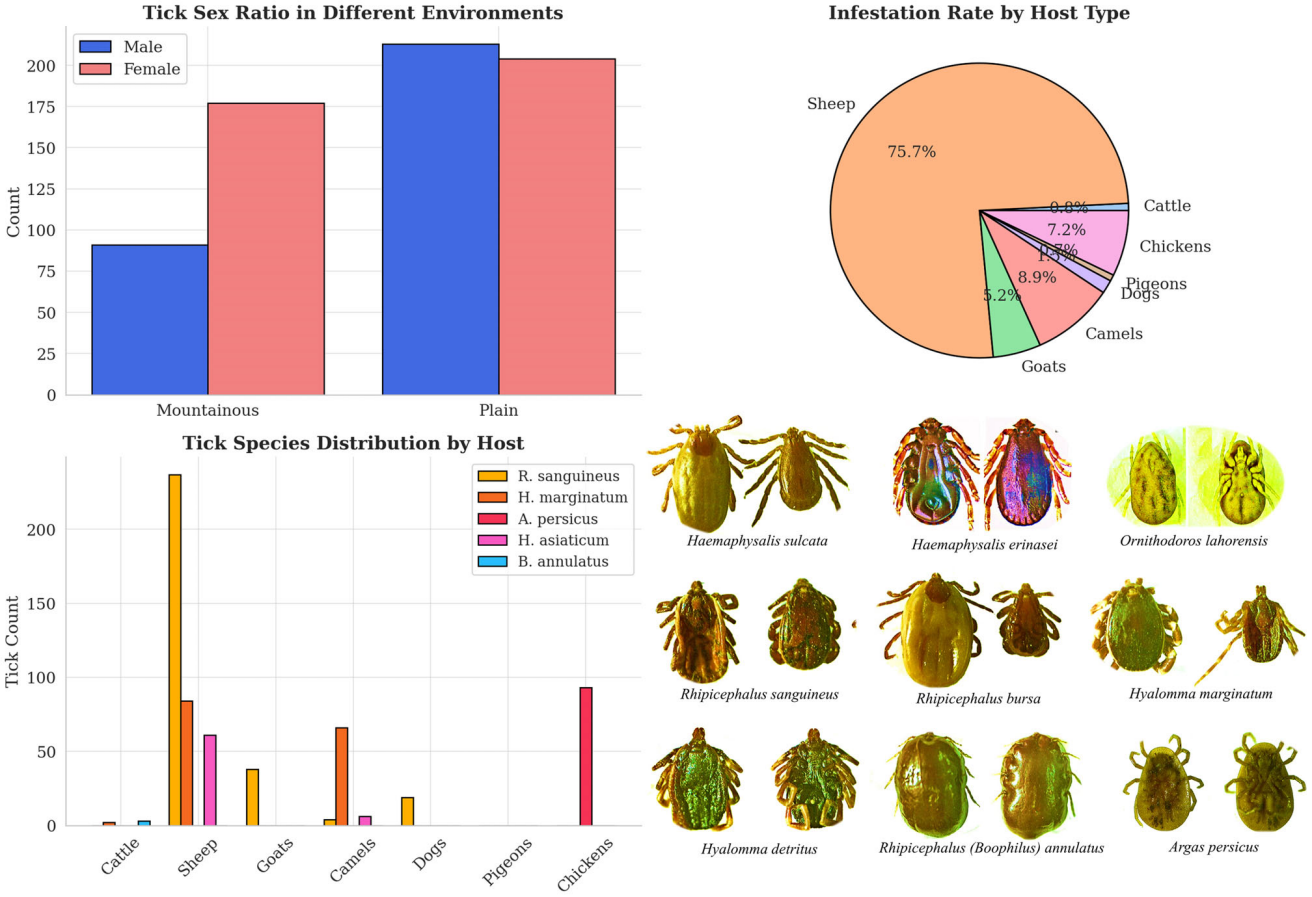
Representative stereomicroscope images of selected tick species identified in Tehran Province, 2019. *Rhipicephalus sanguineus* sensu lato showing hexagonal basis capituli and elongate mouthparts. *Hyalomma marginatum* with narrow mouthparts and distinct leg banding. *Haemaphysalis sulcata* with short mouthparts and rectangular basis capituli. *Boophilus annulatus* characterised by short mouthparts, inornate scutum, and absence of festoons. Diagnostic features are indicated with arrows. Scale bars: 500 µm, 1 mm.

Immediately after removal, ticks were placed into sterile, labelled vials containing 70% ethanol for preservation. In some cases, moistened cotton and perforated caps were used to maintain appropriate humidity. Detailed metadata were recorded for each sample, including host species, sex, age, owner information, village name, collection date, collector name, and the number of ticks collected. Tick identification was conducted in the laboratory using a stereomicroscope and the standard morphological keys provided by Walker et al. (2003), with emphasis on diagnostic features such as mouthparts, scutum shape, and leg segmentation. All identifications were performed by trained entomologists, and 10% of the samples were independently validated by a parasitology expert to ensure taxonomic accuracy. All procedures complied with veterinary ethical guidelines. Verbal consent was obtained from livestock owners, and no animals were harmed during the sampling process (Jongejan et al. 1987). In addition to using Walker et al. (2003) keys, representative individuals of each identified species were photographed under the stereomicroscope. Diagnostic characters, such as mouthpart length and shape, scutum ornamentation, presence of eyes and festoons, and coxal spurs, were annotated to illustrate differences among species. These images are presented in Figure 2. Furthermore, we prepared a comparative table (Table S1) highlighting the principal morphological features distinguishing the most frequently encountered species in Tehran Province. For transparency, the sampling effort by host type and ecological zone is provided in Table S2, which lists the number of animals examined and ticks collected at each step (Abbasi 2025f; Abbasi 2025g; Abbasi 2025h; Abbasi 2025i; Abbasi 2025j).

## 3 Results

### 3.1 Identification and Distribution of Livestock Ticks by Sex

This study gathered 685 hard ticks and 121 soft ticks from a total of 1623 domestic animals, including camels, sheep, cattle, dogs, chickens, and pigeons infected with ticks. Regarding sex segregation among all captured ticks, the results showed that 42.01% of ticks were male, 57.99% female, and 15.01% were soft ticks. A significant difference in tick prevalence was observed among host species, with the highest prevalence found in cattle. No statistically significant difference was found in sex ratio between hosts.

In addition, among 685 hard‐caught ticks (44.37%), 304 were male, and (55.62%) 381 were female. In both mountain and plain climates, *R. sanguineus sensu lato* has the highest sex ratio of hard ticks. Chi‐square analysis showed a statistically significant difference in tick prevalence among different host species (χ^2^ = 204.13, df = 6, *p* < 0.001). Sheep exhibited the highest infestation (60.04%), while cattle showed the lowest (0.62%). Sex ratio differences were significant between mountainous and plain regions (*p* < 0.05), with a higher proportion of females in plains. *R. sanguineus* was the most dominant species across nearly all host types, particularly in dogs and sheep. Chi‐square tests were performed to assess differences in prevalence and sex ratios, with assumptions checked prior to analysis. In cases where expected cell counts were <5, Fisher's exact test was applied. Prevalence values are presented with 95% confidence intervals (CI) to provide a measure of statistical precision (Abbasi 2025k; Abbasi 2025l; Abbasi et al. 2025; Abbasi 2025m; Abbasi 2025n).

### 3.2 Identifying and Determining the Distribution of Ticks by Host Type

Concerning tick‐infested hosts, most ticks were collected from sheep (60.04%), and the lowest were collected from cattle (0.62%). Among the species of captured ticks in the family of hard ticks, the genus *Rhipicephalus (Boophilus)* was collected only from the cattle, the genus *Haemaphysalis* from the sheep and goats, and the genera *Rhipicephalus* and *Hyalomma* were collected from all hosts except for pigeons, and chickens and in the corral wall. In the soft tick family, the genus *Ornithodoros* was collected only from the cage wall, and the genus *Argas* was collected from both pigeons and chickens. Unlike hard ticks, no soft ticks were caught from cattle, sheep, goats, camels, and dogs. Figure 2 provides representative images of the main tick species identified in this study, with diagnostic features indicated by arrows. Table S1 summarises the morphological characters used for differentiation. These visual and tabular aids complement Table 1, allowing for easier comparison and verification of species identifications. Across the entire study, a total of 1623 animals were examined, yielding 806 ticks (685 hard ticks and 121 soft ticks). These totals are consistent across the Abstract, Methods section, and Tables to avoid confusion.

**TABLE 1:** Distribution and identification of tick species by sex and host across different ecological zones in Tehran Province, 2019. Includes total counts and male/female ratios across mountainous and plain regions, and the host‐specific abundance of each species.

Genus/host	Mountainous ♂	Mountainous ♀	Plain ♂	Plain ♀	Cattle	Sheep	Goats	Camels	Dogs	Pigeons	Chickens	Corral wall	Total
*R. sanguineus sensu lato*	74	102	64	58	0	237	38	4	19	0	0	0	596
*H. marginatum*	0	0	91	61	2	84	0	66	0	0	0	0	304
*H. asiaticum*	0	0	26	41	0	61	0	6	0	0	0	0	134
*H. dromedarii*	0	0	17	32	0	17	8	24	0	0	0	0	98
*Hae. sulcata*	0	47	0	0	0	44	3	0	0	0	0	0	94
*H. anatolicum*	0	0	15	6	0	12	0	9	0	0	0	0	42
*Hae. inermis*	12	12	0	0	0	18	6	0	0	0	0	0	48
*Hae. erinacei*	0	9	0	0	0	6	3	0	0	0	0	0	18
*R. bursa*	5	4	0	0	0	5	4	0	0	0	0	0	18
*H. detritum*	0	0	0	6	0	0	0	6	0	0	0	0	12
*B. annulatus*	0	3	0	0	3	0	0	0	0	0	0	0	6
*A. persicus*	—	—	—	—	0	0	0	0	0	0	93	0	93
*O. lahorensis*	—	—	—	—	0	0	0	0	0	0	0	19	19
*A. reflexus*	—	—	—	—	0	0	0	0	0	9	0	0	9
Total	**91**	**177**	**213**	**204**	**5**	**484**	**62**	**115**	**19**	**9**	**93**	**19**	**1491**

*Note*: All tick genera abbreviations are expanded as follows: R.: *Rhipicephalus*, H.: *Hyalomma*, Hae.: *Haemaphysalis*, B.: *Boophilus*, A.: *Argas*, O.: *Ornithodoros*. Host types include cattle, sheep, goats, camels, dogs, pigeons, and chickens. “Corral wall” indicates environmental sampling from livestock enclosures. ♂: male; ♀: female. The total number of hard ticks collected was 685, with 42.01% male and 57.99% female. The most common tick species was *Rhipicephalus sanguineus sensu lato*, the highest infestation rate was in sheep (60.04%), while the lowest was in cattle (0.62%). Soft ticks (*Argas* and *Ornithodoros* genera) were primarily found in pigeons, chickens, and corral walls.

**FIGURE 3:**
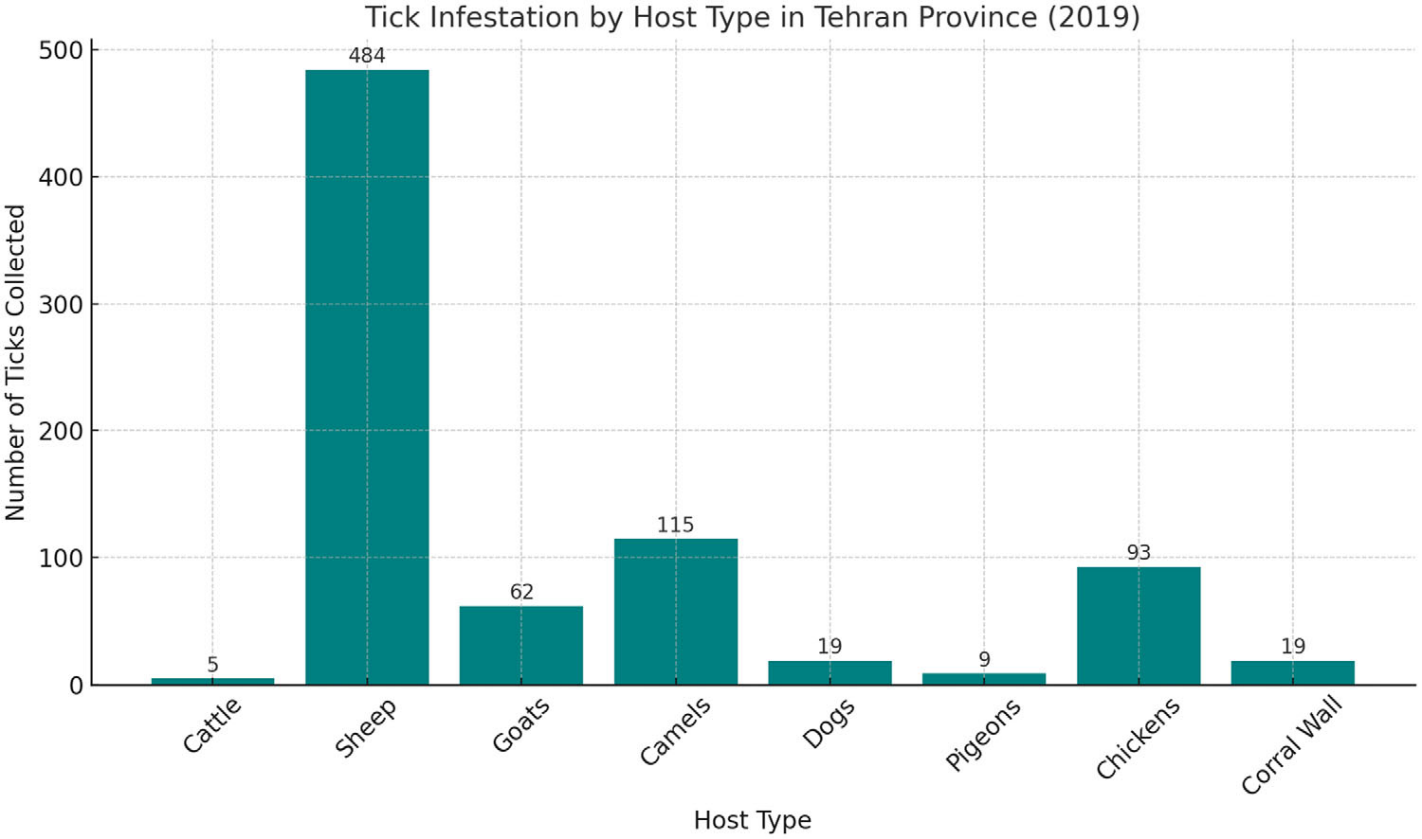
Bar chart (tick sex ratio in different environments): sex‐based distribution of hard ticks in mountainous and plain environments. Pie chart (infestation rate by host type): proportion of tick infestation among different livestock and poultry. Grouped bar chart (tick species distribution by host): host‐specific distribution of tick genera in Tehran Province, collecting ticks from hosts and identifying them using loop (stereomicroscope).

**FIGURE 4:**
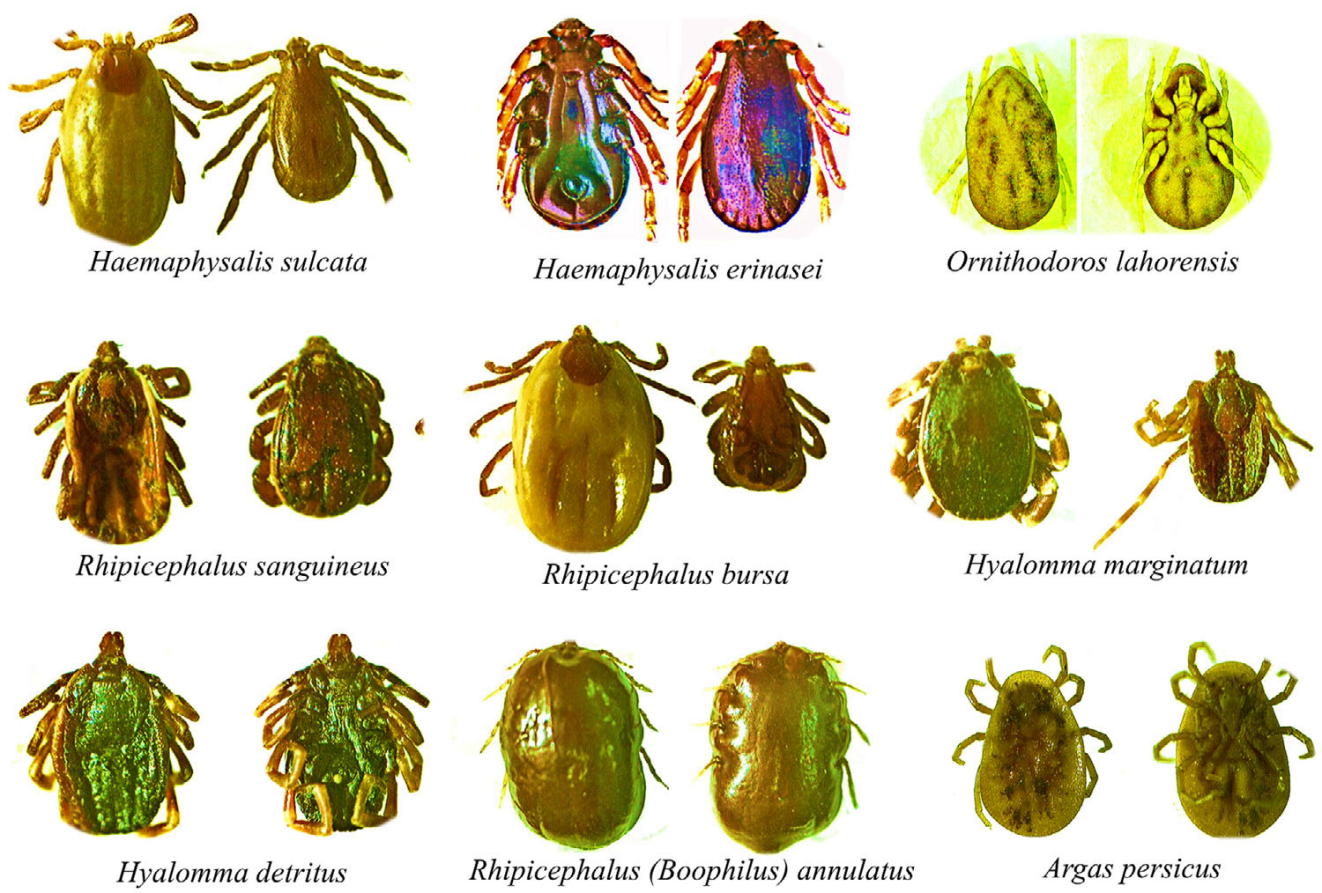
Tick infestation levels by host type in Tehran Province, 2019.

The frequency of tick species caught by host type is such that in cattle, small quantities of two species, *H. Marginatum* and *B. annulatus* were found. In sheep, *Rhipicephalus sanguineus sensu lato*, with 242 specimens, had the highest number, and *Rhipicephalus bursa*, with 5 specimens, had the lowest number. *R. sanguineus sensu lato* is found with the highest accumulation on the body of goats. In camels, the species *of H. Marginatum* had the highest frequency, and *R. sanguineus sensu lato* had the lowest frequency. In dogs, only *R. sanguineus sensu lato* with 19 numbers was found. In pigeons, only *A. reflexus* of the genus *Argas* has been collected. *A. persicus* was collected in significant abundance from the chickens' bodies, and *O. lahorensis* was found only from the corral wall (Table 1 and Figures 3 and 4).

As shown in Figure 3, sheep and goats host the highest variety and load of tick species, particularly *R. sanguineus* sensu lato, which is the most prevalent. This species accounts for over 39% of all identified ticks and shows high adaptability across host types. According to Table 1, tick species distribution varies substantially between regions. In the plains, *Hyalomma marginatum* and *H. asiaticum* are more common, while in the mountains, *Haemaphysalis sulcata* and *R. sanguineus* dominate. Female ticks outnumber males across nearly all species and locations.

## 4 Discussion

The current study results indicate that sex ratios observed in ticks differ among species and even among host populations. Life‐history aspects probably play a significant role in this regard. However, survival analyses under natural conditions are lacking in practically all tick genera, which are crucial to elucidating general patterns. Prospective molecular methods will provide new ways to determine the vast spectrum of possible performers affecting sex ratios. Previous studies demonstrated that sex ratios could depend on the season and area of collection. Also, in arthropods (e.g., ticks), sex ratios could be skewed towards females by reproductive parasites that appertain to this gender for their transmission (transovarial) (Abedi‐Astaneh et al. 2025; De la Fuente et al. 2017; Oliver 1989; Talbalaghi et al. 2024).

Compared to similar studies in Mazandaran, Kurdistan, and Golestan, the present study reveals a unique sex ratio bias and ecological distribution of *R. sanguineus* in Tehran Province, likely due to its semi‐arid climate and host species mix. Additionally, no soft ticks were observed in mammalian hosts, contrasting with *Argas presence* in chickens and pigeons, supporting host specificity theories in Argasidae. Although no new species were discovered, the data offer updated baseline prevalence patterns that can inform surveillance and intervention programs targeting tick‐borne pathogens such as *Babesia* spp. and *Ehrlichia* spp., which are commonly associated with *R. sanguineus* (Abbasi 2024; Abbasi 2025o; Khovand et al. 2022; Khovand et al. 2022; Yakhchali et al. 2012).

Our results showed that the highest rate of tick‐infected livestock was related to sheep, with 60.04% (146 out of 243 sheep) infected, and the lowest rate was related to cattle, with 0.62% (1 out of 161 cattle) infected. This is likely because the majority of studied livestock were sheep. A study conducted in Ghaemshahr city also demonstrated that the highest rate of infected livestock with ticks was observed in sheep at 28.3%, and the lowest in cattle at 20%, which is consistent with our results. The lower infestation rate in cattle may be attributed to the smaller number of cattle included in the study compared to other livestock.

In addition, the dominant fauna species was *Rhipicephalus sanguineus* s.l, which was consistent with the results of the majority of previous research (Nabian et al. 2007; Sofizadeh et al. 2014). One limitation of this study is the lack of control for potential confounding variables such as host age, breed, and environmental or seasonal factors. Future studies should incorporate these variables to gain deeper insights into tick‐host dynamics and epidemiological risks. The higher prevalence in sheep could be attributed to their wool, which provides a suitable microclimate for tick attachment and survival, consistent with reports from other Iranian provinces. The female‐biased sex ratio observed may result from reproductive strategies and environmental sex determination, as noted by Van Oosten et al. (2018). Additionally, ticks may exhibit seasonal sex ratio shifts due to environmental pressures or parasitic manipulation. Our finding that *R. sanguineus* was dominant aligns with its adaptability and widespread distribution in arid to semi‐arid zones. This species is a known vector for Ehrlichia and Babesia, posing zoonotic risks. These insights underscore the importance of integrating sex ratio dynamics and host preferences in designing control programs. These findings, when compared to similar studies across Iran and neighbouring regions, suggest that both host preference and ecological adaptability play key roles in shaping the distribution of tick species. Future research should include molecular confirmation and seasonal tracking to better understand vector–pathogen dynamics (Abbasi and Moemenbellah‐Fard 2024; Abbasi et al. 2025; Abbasi et al. 2025; Dantas‐Torres 2010; Azari‐Hamidian and Harbach 2023).

Climate change has emerged as a critical driver in the shifting dynamics of vector ecology, particularly influencing the distribution, abundance, and seasonal activity of ticks, which are pivotal vectors of numerous zoonotic pathogens. Rising global temperatures, altered precipitation patterns, and extended warm seasons have facilitated the expansion of tick habitats into previously unsuitable regions, including high‐altitude and temperate zones. These climatic shifts enhance tick survival rates, accelerate development cycles, and prolong host‐seeking behaviour, thereby increasing the window of transmission for tick‐borne diseases such as babesiosis, anaplasmosis, and ehrlichiosis. In Iran's semi‐arid regions, including Tehran Province, such ecological changes may amplify the dominance of highly adaptive species like *Rhipicephalus sanguineus* sensu lato, a known vector of *Ehrlichia canis* and *Babesia vogeli*. Additionally, climate‐induced stress on livestock may compromise host immunity, further exacerbating vulnerability to tick infestation and pathogen transmission. The intersection of climate variability and vector biology underscores the need for integrative surveillance systems that incorporate entomological, meteorological, and epidemiological data to anticipate outbreak risks and inform proactive vector control strategies tailored to region‐specific climatic trends (Abbasi 2025p; Abbasi 2025q; Abbasi 2025r; Abbasi 2025s; Abbasi 2025t; Abbasi 2025u).

Insecticide resistance among arthropod vectors, including ticks, poses a significant threat to the efficacy of vector control strategies and the mitigation of tick‐borne diseases in both livestock and human populations. Prolonged and indiscriminate use of acaricides, particularly pyrethroids and organophosphates, has led to the selection of resistant tick populations, reducing the success of chemical interventions. In ticks such as *Rhipicephalus sanguineus* sensu lato, resistance mechanisms—ranging from enhanced metabolic detoxification via cytochrome P450 monooxygenases to target‐site insensitivity through mutations in voltage‐gated sodium channels—have been increasingly reported in endemic regions. These adaptations not only compromise acaricidal efficacy but also facilitate sustained pathogen transmission cycles by allowing vector populations to persist and proliferate. In the context of Tehran Province, where *R. sanguineus* was the predominant species identified, resistance development could severely undermine control efforts against pathogens such as *Babesia spp*. and *Ehrlichia spp*. Therefore, integrated tick management strategies should incorporate regular resistance monitoring, rotation of acaricide classes with different modes of action, and the use of non‐chemical methods such as biological control and habitat modification. Understanding the genetic and ecological basis of acaricide resistance is essential for developing sustainable, evidence‐based approaches to vector management in regions facing rising resistance trends (Abbasi and Daliri 2024; Abbasi and Daliri 2024; Abbasi and Daliri 2024; Abbasi et al. 2025; Abbasi et al. 2024; Abbasi et al. 2023). Vector‐borne diseases such as babesiosis and ehrlichiosis significantly impact livestock health and productivity. This study highlights the dominance of *Rhipicephalus sanguineus* sensu lato in Tehran Province and the importance of understanding tick sex ratios and host preferences. Effective control requires integrated strategies, including surveillance, resistance management, and climate‐adapted interventions, to reduce disease transmission and economic losses (Abbasi 2025v; Abbasi 2025w; Abbasi and Saeedi 2022).

To conclude, the genus and species of dominant ticks in each region are diverse, and the geographical zone and climatic conditions of the area regulate the species and even the sex of active ticks in that province. Therefore, due to the high contamination of sheep, the authorities’ veterinary personnel and ranchers must be in control programs against external livestock parasites (mites) at least twice a year (with a maximum interval of 30 days), in addition to corral pesticide spraying, bathing the animals in the anti‐tick bath. While descriptive, this study presents region‐specific insights into tick sex ratios and host patterns, essential for integrated vector management in Tehran Province. The findings establish a foundation for future risk modelling and molecular surveillance of tick‐borne diseases in central Iran.

## Author Contributions

E.A. has conducted all parts of the study, including design, execution, and writing the manuscript.

## Funding

The author has nothing to report.

## Ethics Statement

This study involved only non‐invasive tick collection from the body surface of livestock. The animals were manually restrained by their owners and examined without sedation or harm. Tick collection was performed by trained personnel under the supervision of local veterinary staff. According to the guidelines of the Shiraz University of Medical Sciences, formal ethical approval was not required for field studies of this type. However, all procedures were conducted in compliance with the national guidelines for the care and use of animals in research (Iranian Council for Animal Ethics, 2010) and adhered to the principles of the Declaration of Helsinki for the ethical treatment of animals.

## Conflicts of Interest

The authors declare no competing interests.

## Supporting information




**Table S1**: Key morphological features for differentiation of major tick species identified in Tehran Province.

**Table S2**. Sampling effort by host type and ecological zone in Tehran Province, 2019.

## Data Availability

All data generated or analysed during this study are included in this published article.
