# Supplementary material for: Prevalence and Identification of Livestock Tick by Sex Ratio and Host in Tehran Province
Source: Vet Med Sci. 2025 Nov 18;11(6):e70702. doi: 10.1002/vms3.70702 (PMC12624458; doi:10.1002/vms3.70702)
Supplement: Supplementary file 1 — Table S1: Key morphological features for differentiation of major tick species identified in Tehran Province. [file VMS3-11-e70702-s002.docx]

**Table S1.** Key morphological features for differentiation of major tick species identified in Tehran Province

| Species | Mouthparts | Scutum | Basis capituli | Leg banding / coxae | Festoons |
| --- | --- | --- | --- | --- | --- |
| *R. sanguineus s.l.* | Elongate | Inornate | Hexagonal posterior margin | Distinct banding absent | Present |
| *H. marginatum* | Long, narrow | Inornate | Rectangular | Banding present | Present |
| *H. asiaticum* | Very long | Inornate | Rectangular | Coxa I with 2 spurs | Present |
| *Hae. sulcata* | Short, broad | Inornate | Rectangular | Coxa IV large, spur present | Present |
| *B. annulatus* | Short | Inornate | Rectangular, wider than long | Coxa I bifid spurs | Absent |
