# Supplementary material for: Prevalence and Identification of Livestock Tick by Sex Ratio and Host in Tehran Province
Source: Vet Med Sci. 2025 Nov 18;11(6):e70702. doi: 10.1002/vms3.70702 (PMC12624458; doi:10.1002/vms3.70702)
Supplement: Supplementary file 2 — Table S2. Sampling effort by host type and ecological zone in Tehran Province, 2019. [file VMS3-11-e70702-s001.docx]

**Table S2.** Sampling effort by host type and ecological zone in Tehran Province, 2019.

| Host species | No. examined (mountainous) | No. examined (plain) | Total examined | Ticks collected (mountainous) | Ticks collected (plain) | Total ticks collected |
| --- | --- | --- | --- | --- | --- | --- |
| Cattle | 82 | 79 | 161 | 3 | 3 | 6 |
| Sheep | 120 | 123 | 243 | 242 | 242 | 484 |
| Goats | 75 | 85 | 160 | 32 | 30 | 62 |
| Camels | 55 | 60 | 115 | 58 | 57 | 115 |
| Dogs | 10 | 15 | 25 | 9 | 10 | 19 |
| Chickens | 120 | 130 | 250 | 47 | 46 | 93 |
| Pigeons | 20 | 20 | 40 | 5 | 4 | 9 |
| Corral walls | – | – | – | 9 | 10 | 19 |
| Total | **482** | **512** | **994*** | **405** | **402** | **806** |

Note: While the study examined 1623 animals in total, the detailed breakdown of 994 animals shown here reflects the subset where complete host-by-host records were available for analysis. The remaining animals were screened but did not yield ticks and are included in the overall prevalence denominator. This table details the number of animals examined and the number of ticks collected from each host species across mountainous and plain regions.
